# IMMUNITY: Mercury Alters Immune System Response in Artisanal Gold Miners

**DOI:** 10.1289/ehp.118-a243

**Published:** 2010-06

**Authors:** Naomi Lubick

**Affiliations:** **Naomi Lubick** is a freelance science writer based in Zürich, Switzerland, and Folsom, CA. She has written for *Environmental Science & Technology, Nature, and Earth.*

The link between mercury and damage to human health is not new: scientists showed several decades ago that an organic form of mercury known as methylmercury causes adverse effects on human neurodevelopment. More recently, scientists have observed associations between immune system disorders and exposure to both organic and inorganic (elemental) mercury.^1^,^2^ A new study of artisanal miners in Brazil could strengthen the link between mercury and immune system problems in humans.^3^ The results of the small-scale cross-sectional study may also apply to other communities exposed to low levels of inorganic mercury, the authors say.

People living downstream from artisanal gold mining sites in Amazonia, Southeast Asia, China, Mongolia, and sub-Saharan Africa are exposed to methylmercury via fish in streams and rivers polluted by small-scale gold mining operations. But public health specialists have long been concerned about more direct exposures to mercury for the miners themselves, who inhale vapors when they burn off mercury they have used to amalgamate gold during the recovery of the precious metal. They also can absorb mercury through their skin as they knead it into the soil sediment to amalgamate the gold—a job Renee Gardner of the Karolinska Institute in Sweden says is often given to children.

Gardner and Ellen Silbergeld of The Johns Hopkins University led a team of researchers attempting to tease out those direct effects of such inorganic mercury exposures. The team started with surveys of artisanal miners in Brazil who worked with either gold, emeralds, or diamonds. The data gathered covered five encampments of artisanal miners, with nearly 250 men and women participating. The researchers collected blood, urine, and hair samples, and screened participants for malaria along with other factors such as residence time at the mining site.

The team measured two immunoglobins (proteins affiliated with autoimmune responses): antinuclear autoantibodies (ANA) and antinucleolar autoantibodies (ANoA). After accounting for infection with malaria, which also stimulates an immune response, their results showed a higher likelihood of ANA and ANoA being detected in miners currently working with gold compared with those mining for emeralds or diamonds, who do not use mercury in their work. However, a small number of gemstone miners—29% of whom reported using mercury in the past—also had detectable levels of ANA, ANoA, or both.

Past research^2^ from some of the team members documented the effects of mercury on activated immune cells in terms of the release of seven cytokines associated with pro- and anti-inflammatory effects in the immune system; that *in vitro* study guided the development of a panel of biomarkers that might characterize human immune sensitivity to mercury compounds. In the current study, mercury exposure correlated with significantly increased levels of three pro-inflammatory cytokines. The authors say these cytokines could serve as biomarkers of mercury-induced immune responses similar to those seen in lupus-like disease (systemic lupus erythematosus is an autoimmune disease that causes chronic inflammation).

“Since these immune changes that we saw were not related to whether or not miners were infected with malaria, the effects were most likely from exposure to mercury,” says coauthor Jennifer Nyland of the University of South Carolina School of Medicine. “We hope [the findings] can be applied to other individuals, as mercury is a global problem. Even if we stopped all small-scale mining in Africa or Brazil, there are still plenty of other sources of mercury”—including measurable mercury contamination in the western United States from historic gold mining.^4^,^5^

“I think the implications are important and may also relate to exposures in the United States from [mercury-bearing] amalgam fillings and religious uses of mercury,” says Philippe Grandjean of the Harvard School of Public Health, who was not involved with the study.

Grandjean says that although the new study points to inorganic mercury as a possible trigger of immune dysfunction, the miners’ long-term mercury exposures were not well documented, and malaria infection could still confound the team’s results. He also points out that although one mining community had high mercury vapor exposure alongside high malaria prevalence, others had low mercury exposure and little malaria; that makes the effects of malaria and mercury exposure difficult to tease out epidemiologically from this one study.

“What we have is a snapshot,” Nyland says of the latest study. Ideally, the team will expand their fieldwork into a larger-scale longitudinal study. They hope to establish partnerships in Brazil to prospectively study ongoing exposure to mercury in artisanal gold miners.

## Figures and Tables

**Figure:**
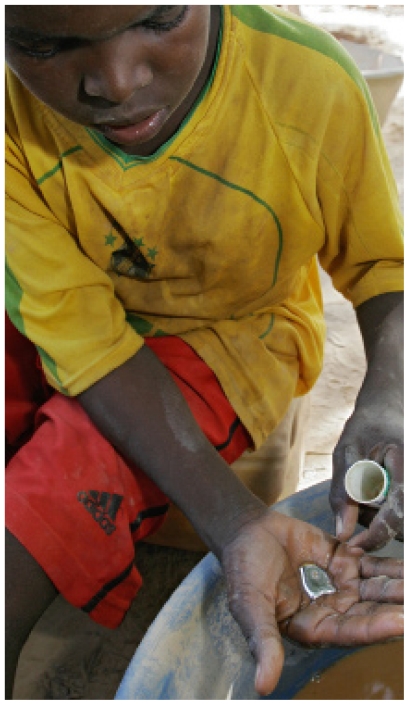
Artisanal miners around the world, including this 13-year-old Senegalese child, use elemental mercury to extract gold particles from soil.
